# Folate-Targeted PEGylated Magnetoliposomes for Hyperthermia-Mediated Controlled Release of Doxorubicin

**DOI:** 10.3389/fphar.2022.854430

**Published:** 2022-03-21

**Authors:** Emílio R. Cintra, Tacio G. Hayasaki, Ailton A. Sousa-Junior, Artur C. G. Silva, Marize C. Valadares, Andris F. Bakuzis, Sebastião A. Mendanha, Eliana M. Lima

**Affiliations:** ^1^ FarmaTec—Laboratory of Pharmaceutical Technology, School of Pharmacy, Federal University of Goias, Goiania, Brazil; ^2^ Toxin—Laboratory of Education and Research in In Vitro Toxicology, School of Pharmacy, Federal University of Goias, Goiania, Brazil; ^3^ Physics Institute, Federal University of Goias, Goiania, Brazil; ^4^ CNanoMed—Nanomedicine Integrated Research Center, Federal University of Goias, Goiania, Brazil

**Keywords:** magnetoliposomes, doxorubicin, magnetic hyperthermia, folic acid, B16F10, MCF7

## Abstract

Doxorubicin (DOX) is a chemotherapeutic agent commonly used for the treatment of solid tumors. However, the cardiotoxicity associated with its prolonged use prevents further adherence and therapeutic efficacy. By encapsulating DOX within a PEGylated liposome, Doxil^®^ considerably decreased DOX cardiotoxicity. By using thermally sensitive lysolipids in its bilayer composition, ThermoDox^®^ implemented a heat-induced controlled release of DOX. However, both ThermoDox^®^ and Doxil^®^ rely on their passive retention in tumors, depending on their half-lives in blood. Moreover, ThermoDox^®^ ordinarily depend on invasive radiofrequency-generating metallic probes for local heating. In this study, we prepare, characterize, and evaluate the antitumoral capabilities of DOX-loaded folate-targeted PEGylated magnetoliposomes (DFPML). Unlike ThermoDox^®^, DOX delivery via DFPML is mediated by the heat released through dynamic hysteresis losses from magnetothermal converting systems composed by MnFe_2_O_4_ nanoparticles (NPs) under AC magnetic field excitation—a non-invasive technique designated magnetic hyperthermia (MHT). Moreover, DFPML dismisses the use of thermally sensitive lysolipids, allowing the use of simpler and cheaper alternative lipids. MnFe_2_O_4_ NPs and DFPML are fully characterized in terms of their size, morphology, polydispersion, magnetic, and magnetothermal properties. About 50% of the DOX load is released from DFPML after 30 min under MHT conditions. Being folate-targeted, *in vitro* DFPML antitumoral activity is higher (IC_50_ ≈ 1 μg/ml) for folate receptor-overexpressing B16F10 murine melanoma cells, compared to MCF7 human breast adenocarcinoma cells (IC_50_ ≈ 4 μg/ml). Taken together, our results indicate that DFPML are strong candidates for folate-targeted anticancer therapies based on DOX controlled release.

## Introduction

The National Cancer Institute (NCI) defines cancer as “a disease in which some of the body’s cells grow uncontrollably and spread to other parts of the body” (National Cancer Institute, 2021b). A more formal definition was presented by Hanahan and Weinberg (Hanahan and Weinberg, 2000, 2011), in which ten hallmark capabilities of cancer are compiled: 1) sustaining proliferative signaling; 2) evading growth suppressors; 3) resisting cell death; 4) enabling replicative mortality; 5) inducing angiogenesis; 6) activating invasion and metastasis; 7) deregulating cellular energetics; 8) avoiding immune destruction; 9) genome instability and mutation; and 10) tumor-promoting inflammation. For 2021, the NCI estimated almost two million new cancer cases and more than 600,000 deaths in the Unites States (American Cancer Society, 2021). Worldwide, the estimates for 2020 totalized about 19 million new cases and 10 million deaths (Sung et al., 2021). According to estimates of the World Health Organization (WHO), cancer is the first or second leading cause of death in about 60% of the countries (Sung et al., 2021). In 2020, the worldwide spending in oncology surpassed 167 billion U.S. dollars (Mikulic, 2021), which highlights the need for new approaches to cancer diagnostics and treatment.

Anticancer treatments depend on the type and progression of the disease. The most conventional treatments are surgery, radiotherapy, and chemotherapy, or a combination of these. Other therapies available include hormonotherapy, immunotherapy, stem cell transplant, and targeted therapy (National Cancer Institute, 2021a). Among new therapies under research and/or clinical trials, nanotechnology-based therapies involving site-specific drug delivery systems (DDS) have gained momentum through the past decades. Particularly, liposomal DDS figure up as a mature technology, with a doxorubicin liposomal formulation (Doxil^®^) being the first FDA-approved nanomedicine (Barenholz, 2012a).

Doxorubicin (DOX) is one of the most commonly used chemotherapeutic agents for treating solid tumors, such as soft tissue sarcomas. However, the high occurrence of cardiotoxic side effects associated with its prolonged use prevents further adherence and therapeutic efficacy (Shi et al., 2011). Doxil^®^ tackled this issue by encapsulating DOX within a PEGylated liposome via a transmembrane ammonium sulfate gradient-driven remote loading method (Barenholz, 2012a). Nonetheless, DOX release at the desired site of action would be substantially dependent on the ratio $k_{off}/k_{c}$ , where $k_{off}$ represents DOX release rate from the liposome, and $k_{c}$ is its blood clearance rate (Barenholz, 2012a). PEGylation slows clearance, consequently increasing this ratio, but other strategies available could be adopted, aiming at a more controlled release of DOX, especially at tumor sites (Barenholz, 2012a; 2012b).

ThermoDox^®^ approached this issue by developing a DOX-loaded lysolipid thermally sensitive liposome (LTSL) (Poon and Borys, 2009). When submitted to hyperthermia conditions (i.e., temperatures within 39 and 43°C), this thermo-sensitive liposome would be lysed, releasing its DOX contents in a site-specific manner. According to its manufacturer, the amount of DOX released would be 25 times greater than by intravenous infusion and 5 times greater than that released by Doxil^®^ (Celsion Corporation ThermoDox^®^, 2021). Nevertheless, the thermo-sensitive lipids present in its composition can significantly inflate production costs and, consequently, therapy prices. Moreover, ThermoDox^®^ commonly rely on radiofrequency (RF)-induced heating, which involves the introduction of invasive metallic probes into the tumor, eventually preventing extensive applications (May and Li, 2013; Swenson et al., 2015).

In this study, we prepare, characterize, and evaluate the antitumoral capabilities of DOX-loaded folate-targeted PEGylated magnetoliposomes (DFPML). Like Doxil^®^, DOX-encapsulation in DFPML decreases its cardiotoxicity potential, whereas PEGylation prolongs its half-life in blood, increasing the probability of its retention in tumor areas. Like ThermoDox^®^, DOX-release can be controlled in a heat-induced manner. Unlike ThermoDox^®^, however, heat is released through dynamic hysteresis losses from magnetothermal converting systems composed by MnFe_2_O_4_ nanoparticles (NPs) excited by an alternating current (AC) magnetic field. This noninvasive technique is known as magnetic hyperthermia (MHT) and can be used to treat tumors anywhere in the body (Rodrigues et al., 2020). Moreover, production costs are significantly lowered since thermo-sensitive lipids are not required for DFPML manufacturing. Instead, DOX-release is mediated by heat-induced pore formation throughout the liposome bilayer (enhanced permeation). In addition, in contrast with both Doxil^®^ and ThermoDox^®^, DFPML are functionalized with folate, improving their retention specifically in folate receptor-rich areas. Since most tumors rely on folic acid (folate) for disease progression, DFPML are strong DDS candidates for folate-targeted anticancer therapies based on DOX controlled release.

## Materials and Methods

### Synthesis of Manganese Ferrite Nanoparticles (MnFe_2_O_4_ NPs)

Manganese ferrite (MnFe_2_O_4_) nanoparticles were synthesized via co-precipitation, as previously described (Sousa-Junior et al., 2020). Briefly, 90 ml of methylamine (CH_3_NH_2_, Sigma-Aldrich) were first diluted in 400 ml of ultrapure water (MilliQ) under magnetic stirring and heating until boiling. Then, 50 ml of a 0.5 M manganese chloride (MnCl_2_, Sigma-Aldrich) solution and 50 ml of a 1 M iron-III chloride (FeCl_3_, Sigma-Aldrich) solution were added, initiating the synthesis reaction, governed by the equation:

$${\text{Mn}}^{+2}{+2\text{Fe}}^{+3}{+8\text{OH}}^{-}\to {\text{MnFe}}_{2}{\text{O}}_{4}{+4\text{H}}_{2}\text{O}$$

In the following 30 min, a black precipitate containing the magnetic NPs was gradually formed, which was then magnetically separated, washed three times, and re-suspended in ultrapure water.

### MnFe_2_O_4_ NPs Coating With Citrate, Phosphate, or Dextran

To obtain a stable magnetic colloid, the MnFe_2_O_4_ NPs must be surface-coated with either ions or molecules, providing either electrostatic and/or steric repulsion between the magnetic cores. Three different coatings were tested in our studies, as follows.

Citrate coating was obtained by adding 1 mol of sodium citrate (Na_3_C_6_H_5_O_7_, Sigma-Aldrich) for every 10 mol of iron-III ions initially added for the synthesis reaction, as previously reported (Sousa-Junior et al., 2020). The dispersion became turbid and brownish. After 10 min under magnetic stirring and controlled heating (80°C), the colloid is left for cooling at room temperature. The coated NPs are then magnetically separated, washed three times in acetone, and resuspended in ultrapure water.

Phosphate coating was obtained by adding 25 ml of a 0.15 M sodium triphosphate solution (Na_5_P_3_O_10_, Sigma-Aldrich) for every 10 g of iron-III chloride initially added for the synthesis reaction. After 60 min of magnetic stirring, the colloid pH was raised to 8. To remove non-adsorbed phosphate ions, the dispersion was submitted to dialysis (12–14 kDa-pore membrane) under magnetic stirring for 72 h. The 1,000 ml of ultrapure water initially surrounding the dialysis bag were replaced every 12 h.

Dextran coating was obtained by adding 9.2 g of dextran ([C_6_H_10_O_5_]_n_, Sigma-Aldrich) to every 18.4 g of MnFe_2_O_4_ NPs (1:2 mass ratio) resuspended in 50 ml of ultrapure water. After 30 min of magnetic stirring, the dispersion was submitted to tumbling homogenization for 24 h. To remove non-adsorbed dextran molecules, the colloid was also submitted to dialysis (12–14 kDa-pore membrane) under magnetic stirring for 72 h, with water replacement every 12 h.

### Characterization of MnFe_2_O_4_ NPs by TEM, XRD, VSM, and MHT

To verify the morphology and the size distribution of the MnFe_2_O_4_ NPs, selected aliquots were submitted to transmission electron microscopy (TEM, Jeol, JEM-2100, Thermo Scientific). TEM images were then processed through ImageJ (NIH, USA), providing both MnFe_2_O_4_ NPs diameter distribution (*via* nanoparticle counting) and selected interplanar distances (spacing between crystalline planes within a single NP crystal). To assess the NPs size and crystallinity, selected samples were submitted to X-ray diffraction (XRD, XRD-6000, Shimadzu).

To determine the colloid magnetic concentration, the specific magnetizations of both dried (powder) and fluid aliquots of selected samples were determined by Vibrating Sample Magnetometry (VSM, EV9 Magnetometer, ADE Magnetics), with the magnetic concentration being approximately equal to:

$$x\approx {\text{ρ}}_{s}\frac{{\text{σ}}_{fluid}}{{\text{σ}}_{powder}}$$ (1)

where x represents the magnetic concentration of the colloid (g/ml), ${\text{ρ}}_{s}$ represents the density (g/ml) of the solvent in which the coated NPs were resuspended (ultrapure water, in our case), and σ denotes the specific saturation magnetization (emu/g).

To determine the MnFe_2_O_4_ NPs efficiency as magnetothermal converting agents, the specific loss power (SLP, given in W/g) of selected samples was determined at the AC magnetic field frequency of 334 kHz for various field amplitudes (within 100–342 Oe) made available by a MagneTherm (NanoTherics). SLPs were determined as follows (Carrião and Bakuzis, 2016):

$$SLP=\frac{\text{ρ}c}{x}{\left(\frac{dT}{dt}\right)}_{t\to 0}$$ (2)

where ρ and c represent, respectively, the density (g/ml) and the specific heat (Jg^−1^°C^−1^) of the sample (both assumed to be approximately equal to those of the solvent, ultrapure water in our case), whereas x denotes the sample magnetic concentration (g/ml), and ${\left(dT/dt\right)}_{t\to 0}$ is the rate at which the sample temperature T (°C) changes in time t (s), for initial MHT times.

### Magnetoliposomes (ML)

MnFe_2_O_4_ NPs-loaded liposomes (magnetoliposomes) were prepared by lipid film hydration followed by co-extrusion with diluted ferrofluid samples (Mayer et al., 1986). Briefly, soy phosphatidylcholine (PC, Lipoid) and cholesterol (CHOL, Sigma-Aldrich) were dissolved in chloroform (3 ml, 2:1 molar ratio, PC:CHOL). Next, chloroform was completely removed at room temperature and low pressure using a rotary evaporator (IKA), forming a lipid film on the flask’s inner surface. Then, 4 ml of diluted ferrofluid samples (citrate-, phosphate-, or dextran-coated MnFe_2_O_4_ NPs in 300 mM (NH_4_)_2_SO_4(aq)_ buffer, pH = 3, 1:3 v:v, ferrofluid:buffer) were used to hydrate the lipid film. The resulting dispersion was then extruded 8 times (bench extruder, Northern Lipids) under pressurized nitrogen flow (N_2_, 150 psi) through porous polycarbonate membranes (Whatman) with average pore diameters of 600 nm. Finally, the extruded dispersion was submitted to eight additional extrusion cycles through 200 nm-pore membranes, followed by eight extrusion cycles through 100 nm-pore membranes. The external medium (pH = 3) was then replaced by HEPES buffer (pH = 7.4), and the resulting dispersion was submitted to size exclusion chromatography (SEC, Sephadex G50 column) to separate non-encapsulated MnFe_2_O_4_ NPs from the actual magnetoliposomes. The final formulation was characterized by VSM, MHT, and dynamic light scattering (DLS, Zetasizer, Malvern).

### Folate-Targeted PEGylated Magnetoliposomes (FPML)

To prepare PEGylated magnetoliposomes targeting folate receptors, we first proceeded with the synthesis of DSPE-PEG-FA, where DSPE-PEG stands for 1,2-distearoyl-sn-glycero-3-phosphoethanolamine-N-[amino (polyethylene glycol)-2000], and FA designates folic acid. DSPE-PEG-FA was synthesized as previously reported (Chan et al., 2007; Zhang and Yao, 2012; Peres-Filho et al., 2018).

Briefly, 100 mg of FA were dissolved in a 4 ml solution of anhydrous DMSO (dimethyl sulfoxide, Sigma Aldrich) and 1.25% (50 μL) of triethylamine (Sigma Aldrich), under agitation, in a light-protected and anhydrous environment, for 12 h. Next, dicyclohexyl-carbodiimide (DCC, Sigma Aldrich) and N-hydroxysuccinimide (NHS) were added, and agitation, under the same experimental conditions, was kept for additional 18 h. The molar ratio FA:DCC:NHS was 1:1:2. During this step, a precipitate (dicyclohexylurea, DCU) was gradually formed, which was then removed through a 220 nm syringe filter (Millex). Then, 400 mg of DSPE-PEG (Avanti) were added to the filtered FA solution, and the resulting solution was kept under agitation, in a light-protected environment, for 12 h. Finally, 50 ml of ultrapure water were added, and the diluted product was centrifuged (MiniSpin Plus, Eppendorf), to remove traces of water-insoluble reagents. To completely remove DMSO and free FA, the DSPE-PEG-FA solution was submitted to dialysis (3.5 kDa-pore membrane), twice against saline and three times against ultrapure water. The dialysate DSPE-PEG-FA was frozen (−20°C) and lyophilized (Thermo Electron Corporation), at low pressure and low temperature, for 36 h. The synthesis of DSPE-PEG-FA was confirmed by Fourier Transform Infrared Spectroscopy (FTIR, Varian 640-IR).

To functionalize the pre-formed magnetoliposomes, DSPE-PEG-FA molecules were post-inserted following a previously reported technique (Uster et al., 1996; Nakamura et al., 2012). Briefly, 12.6 mg of DSPE-PEG and 1.6 mg of DSPE-PEG-FA were dissolved in 1 ml of chloroform within a glass flask. The solvent was then forced to evaporate under N_2_ flow. Next, 1 ml of the magnetoliposomes formulation was added to the DSPE-PEG + DSPE-PEG-FA film. The dispersion was submitted to slow orbital shaking (IKA, KS 400) for 24 h. Similarly, control samples (non-folate targeted PEGylated magnetoliposomes, PML) were also prepared. Both control and study samples were prepared in triplicate.

### DOX-Loaded Folate-Targeted PEGylated Magnetoliposomes (DFPML)

To encapsulate doxorubicin (DOX) within the pre-formed folate-targeted PEGylated magnetoliposomes, a method involving both pH and [NH_4_^+^] gradients was adopted, as previously reported (Barenholz, 2001; 2012b). In summary, these gradients favor DOX influx and subsequent reaction with sulfate ions within the liposome’s aqueous core, forming an insoluble salt (DOX-SO_4_^−2^) that precipitates and thus remains trapped within the liposome (Haran et al., 1993; Zucker et al., 2009). Briefly, 2 mg of lyophilized DOX hydrochloride were added to 1 ml of the FPML formulation and kept at 4°C for 12 h. The resulting DFPML formulation was characterized by nanoparticle tracking analysis (NTA, Nanosight), DLS, VSM, and MHT. In addition, cryo-TEM (FEI Tecnai F20 Twin) was performed for a non-magnetic version of the nanocarrier but DOX-loaded following the same method used to prepare the complete DFPML formulation.

DOX encapsulation efficiency (EE) was determined by the following equation:

$$\text{EE}\left(\text{\%}\right)=100\text{×}\frac{\text{encapsulated}\;\text{DOX}\;\text{mass}}{\text{initial}\;\text{DOX}\;\text{mass}}$$ (3)

### MHT-Mediated Controlled Release of DOX

To study the magnetic hyperthermia-mediated controlled release of DOX, 500 µL of DOX-loaded magnetoliposomes (either folate-target or not) were submitted to MHT using a MagneTherm (NanoTherics). Once the AC magnetic field was applied (342 Oe, 334 kHz), the sample temperature was raised to 43°C within about 10 min. The magnetic field amplitude was then controlled, to keep the sample temperature at 43°C for 30 min.

Next, the samples were submitted to SEC (Sephadex G50) to separate MHT-mediated released DOX molecules from those still entrapped by the liposomal formulation. Then, liposomal DOX aliquots were lyophilized and resuspended in 500 μL of ultrapure water. To enable DOX extraction, 2 ml of methanol were added, and the sample was sonicated at room temperature for 20 min. Next, the sample was poured into a 5 ml volumetric flask, and the flask was filled up to 5 ml with methanol. This volume was appropriately partitioned into falcon tubes, which were centrifuged (2000×g) for 10 min. Finally, the supernatants were collected for the quantitation of the extracted DOX via high-performance liquid chromatography (HPLC). DOX release (%) was obtained as follows:

$$released\;DOX\;\left(\%\right)=100\times \left(1-\frac{\left[DOX\;after\;MHT\right]}{\left[DOX\;before\;MHT\right]}\right)$$ (4)

### DOX Quantitation via HPLC-UV

DOX quantitation was accomplished via high-performance liquid chromatography (HPLC, Varian Pro Star) followed by absorbance determination within the UV spectrum.

Free DOX maximum absorbance, obtained at 233 nm, was first determined by UV-Vis spectrophotometry (Varian Cary 50). The UV-Vis detector coupled to the HPLC system available was then set up to detect absorbance values at 233 nm.

Chromatographic runs were performed through a Zorbax SB-C18 column (250 mm × 4.6 mm, 5 μm, Agilent), with the mobile phase being composed of acetonitrile (ACN) and acidified water (trifluoroacetic acid, TFA, 1%) at the volumetric ratio 38:62 (ACN:H_2_O). The automated injection volume was 20 μL, the run duration was set to 7 min, and the flow rate was equal to 1 ml/min. The analytical method was validated, showing selectivity, linearity (1–50 μg/ml range, *r* ^2^ > 0.99), precision (RSD% values <5%), and accuracy (>95%). While the detection limit was established at 0.25 μg/ml, the quantitation limit was defined at 1 μg/ml (RSD% values <5%). This optimized analytical method was based on previously reported methods for DOX quantitation (Al-Abd et al., 2009; Urva et al., 2009; Bermingham et al., 2010).

### *In vitro* Cytotoxicity of DFPML

B16F10 murine melanoma and MCF7 human mammary adenocarcinoma cell lines were grown in DMEM (Dulbecco’s Modified Eagle’s Medium), supplemented with 10% of FBS (fetal bovine serum) and 1% of penicillin-streptomycin, in a 5% CO_2_ atmosphere, at 37°C. Cells were allowed to grow for 24 h, and then exposed to different concentrations of control and study formulations. Next, cells were incubated for additional 24 h prior to MTT viability assays. Half-maximal inhibitory concentrations (IC_50_) were determined for each tested formulation via non-linear regression of the cytotoxicity data.

### *In vitro* Temperature-Induced Antitumoral Activity of DFPML

The *in vitro* antitumoral activity of control and study formulations was investigated under two different treatment temperatures. B16F10 cells (96-well plate, 10,000 cells/well, RPMI-1640 medium w/o folate) were exposed (24 h after plating) to the IC_50_ concentrations of the tested formulations, under either 37°C or 43°C, for 1, 2, or 4 h. Cells were kept in a 5% CO_2_ atmosphere during treatment. Experiments were carried out in triplicate.

### Statistical Analysis

Measurements were expressed as mean value ± SD. Statistical analyses were done via analysis of variance (ANOVA) and/or student’s *t*-test using GraphPad Prism. Statistically significant differences were considered for *p* < 0.05.

## Results

### Manganese Ferrite Nanoparticles (MnFe_2_O_4_ NPs) Characterization

**FIGURE 1:**
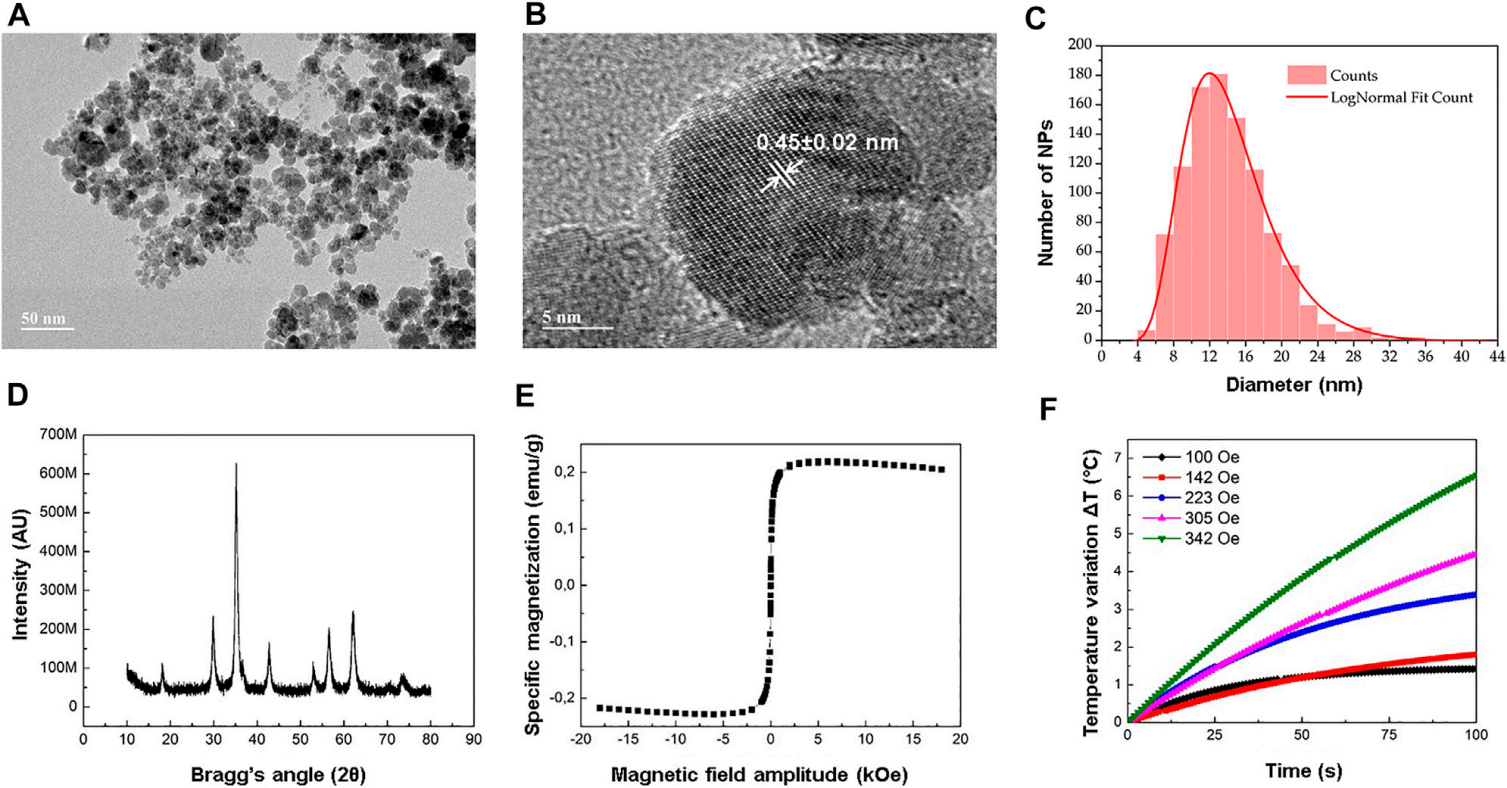
MnFe_2_O_4_ NPs characterization. **(A)** Typical TEM image of an aqueous (ammonium sulfate buffer) dispersion of MnFe_2_O_4_ NPs dried on the surface of a TEM sample holder (grid). Scale: 50 nm. **(B)** High resolution TEM image of a typical NP, evidencing its crystallographic planes and roughly spherical morphology. Scale: 5 nm. **(C)** Frequency count ( N = 1,000) of TEM-imaged NPs. Mean diameter = 14.4 ± 5.2 nm, obtained via a log-normal fit of the diameter distribution. **(D)** XRD spectrum of powder-dried NPs. Mean diameter = 13.5 nm, obtained via Debye–Scherrer equation. **(E)** Typical magnetization profile of NPs displaying superparamagnetic behavior (no hysteresis), obtained via VSM. Magnetic concentration = 4.5 mg/ml, calculated *via* Eq. 1. **(F)** MHT profiles used to calculate the NPs magnetothermal conversion efficiency (i.e., their specific loss power, SLP) *via* Eq. 2, for different AC magnetic field amplitudes. AC magnetic field frequency = 334 kHz. See Supplementary Table S1 for the corresponding SLP values.

Figures 1A,B show representative transmission electron microscopy (TEM) images of the MnFe_2_O_4_ NPs synthesized for this work. Regarding their morphology, MnFe_2_O_4_ NPs synthesized *via* co-precipitation are roughly spherical. From the log-normal fit (Figure 1C) parameters, their mean diameter was estimated as 14.4 ± 5.2 nm.

Figure 1D shows the X-ray diffractogram (XRD) for a representative powder sample of the original colloidal aqueous dispersion of MnFe_2_O_4_ NPs. The crystallite mean diameter, obtained via XRD, was 13.5 nm.

Figure 1E shows the magnetization curve, obtained via vibrating sample magnetometry (VSM), for the dextran-coated MnFe_2_O_4_ NPs ferrofluid. Under VSM conditions, the magnetic NPs display a quasi-static superparamagnetic behavior, superimposed to a weak diamagnetic signal from the solvent. This signal causes a slight clockwise rotation of the magnetization curve about the origin. Discounted this diamagnetic signal, the specific saturation magnetization estimated for the fluid was 0.23 emu/g ( ${\text{σ}}_{\text{fluid}}$ ), whereas a powder version of this sample showed specific saturation magnetization of 51.11 emu/g ( ${\text{σ}}_{\text{powder}}$ , Supplementary Figure S1). Since the NPs were dispersed in (NH_4_)_2_SO_4(aq)_ buffer, with density $\text{ρ}\approx {\text{ρ}}_{H_{2}O}$ , the ferrofluid magnetic concentration was determined *via* Eq. 1 as being 4.5 mg/ml.

Figure 1F shows multiple magnetic hyperthermia (MHT) profiles for the dextran-coated MnFe_2_O_4_ NPs ferrofluid. Higher MHT-induced temperatures could be achieved by gradually increasing the AC magnetic field amplitude from 100 Oe to 342 Oe, whereas the field frequency was fixed at 334 kHz. Similarly, the specific loss power (SLP), calculated *via* Eq. 2, increased with the field amplitude (Supplementary Table S1), with the SLP values proportional to the square of the AC magnetic field amplitude (Supplementary Figure S2).

### DOX-Loaded Folate-Targeted PEGylated Magnetoliposomes (DFPML) Characterization

**FIGURE 2:**
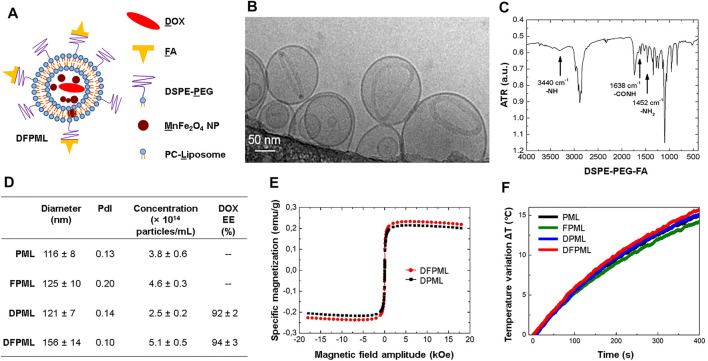
DOX-loaded folate-targeted PEGylated magnetoliposomes (DFPML) characterization. **(A)** Schematic view of DFPML, with D recalling doxorubicin (DOX); F, folic acid (or the corresponding folate moiety); P, the PEG_2000_ composing the DSPE-PEG molecule; M, the MnFe_2_O_4_ magnetic NPs; and L, the phosphatidylcholine-based liposome (PC-Liposome). **(B)** Cryo-TEM representative image of a non-magnetic aliquot of DFPML (MnFe_2_O_4_ NPs absent, emphasis on the water-insoluble DOX salt entrapped within the liposome aqueous core). **(C)** FTIR spectrum of the synthetic molecule DSPE-PEG-FA, responsible for the folate-targeting property of DFPML. **(D)** Summary of size and number distribution properties of DFPML (study) and other (control) samples, either not loaded with DOX (PML and FPML) or non-folate-targeted (DPML). DOX encapsulation efficiency (EE, calculated *via* Eq. 3) for DOX-loaded samples (DPML and DFPML). **(E)** Magnetization profiles for DFPML and DPML, from which their magnetic concentrations could be determined *via* Eq. 1 (4.5 mg/ml and 4.2 mg/ml, respectively). **(F)** MHT profiles used to calculate the SLP values for PML, FPML, DPML, and DFPML. AC magnetic field = 342 Oe, 334 kHz. See Supplementary Table S2 for the corresponding SLP values.

Figure 2A is a schematic view of the doxorubicin-loaded folate-targeted PEGylated magnetoliposomes (DFPML) prepared for this study. To be noted that not all of the DSPE-PEG molecules were functionalized with the folate group (denoted FA, to emphasize that it is derived from the deprotonation of a folic acid molecule), as described in the Materials and Methods. Moreover, although most of the water-stable MnFe_2_O_4_ NPs are expected to be found within the liposome aqueous core, some of them might as well be entrapped within the liposome lipidic phase, as a byproduct of the extrusion process. Also, although doxorubicin (DOX) base is a hydrophobic drug, and hence would normally be carried within the liposome lipidic phase, the DFPML actually carry a water-insoluble DOX-derived salt, crystallized within the liposome aqueous core, as shown in Figure 2B. The cryo-TEM image in Figure 2B was taken for a non-magnetic version of the nanocarriers. Nevertheless, they were DOX-loaded following the same method used to prepare the complete DFPML formulation, as described in the Materials and Methods.

Figure 2C shows a Fourier Transform Infrared Spectroscopy (FTIR) analysis of DSPE-PEG-FA, a synthetic molecule, created for the functionalization (active tumor-targeting) of the DFPML. Supplementary Figure S3 shows the FTIR spectrum for DSPE-PEG alone, whereas Supplementary Figure S4 shows the FTIR spectrum for folic acid (FA) alone. Analyzing the highlighted peaks in these three spectra, which correspond to vibrations (stretching) of the functional groups pointed out in the figures, FTIR results suggest that DSPE-PEG and FA indeed react to form DSPE-PEG-FA molecule.

Figure 2D summarizes the information about the nanocarriers: mean hydrodynamic diameter and PdI (polydispersity index), both acquired via dynamic light scattering (DLS); and concentration (number of particles/mL), obtained via nanoparticle tracking analysis (NTA), for different samples. PEGylated magnetoliposomes (denoted PML) do not carry DOX and are not folate-targeted, whereas folate-targeted PEGylated magnetoliposomes (FPML) also do not carry DOX but are obviously folate-targeted. Their corresponding DOX-loaded versions are represented by the acronyms DPML and DFPML, respectively. All of the samples displayed low PdI, attesting that their preparation yielded virtually monodispersed samples. Moreover, the mean hydrodynamic diameters of PML, FPML, and DPML control samples are not significantly different from each other (all in the vicinity of 120 nm), although the mean hydrodynamic diameter of DFPML samples was significantly higher (30%). Figure 2D also brings DOX encapsulation efficiency (EE) for DPML and DFPML samples, both above 90%, calculated *via* Eq. 3.

Figure 2E shows the magnetization curve, obtained via VSM, for DPML and DFPML samples. Similar magnetization profiles were obtained for both samples, with specific saturation magnetization of 0.21 emu/g for DPML and 0.23 emu/g for DFPML. Since the liposome aqueous core was composed of (NH_4_)_2_SO_4(aq)_ buffer, with density $\text{ρ}\approx {\text{ρ}}_{H_{2}O}$ , the magnetic concentrations of DPML and DFPML were determined *via* Eq. 1 as being 4.2 mg/ml and 4.5 mg/ml, respectively (with ${\text{σ}}_{\text{powder}}$ = 51.11 emu/g, see Supplementary Figure S1).

Figure 2F shows the magnetic hyperthermia (MHT) profiles for PML, FPML, DPML, and DFPML samples, under AC magnetic field with amplitude 342 Oe and frequency 334 kHz. In agreement with their magnetic characterization (via VSM, with similar magnetization profiles), all samples exhibited similar MHT heating profiles, and consequently similar SLP values, all around 56.0 W/g, as shown in Supplementary Table S2.

### MHT-Mediated Controlled Release of DOX

**FIGURE 3:**
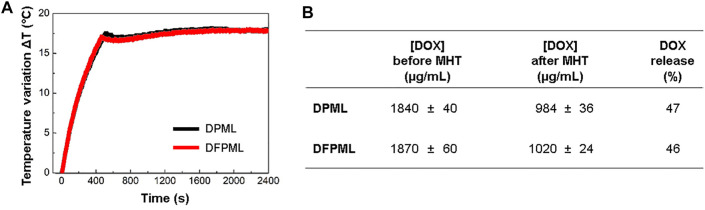
MHT-mediated controlled release of DOX. **(A)** MHT profiles of DOX-loaded nanocarriers (DPML and DFPML). AC magnetic field frequency of 334 kHz, and amplitude controlled to keep the sample temperature at 43°C for 30 min **(B)** DOX concentration in DPML and DFPML before and after MHT. Percentage of released DOX from DPML and DFPML, calculated *via* Eq. 4.

Figure 3A shows the MHT profiles for DPML and DFPML samples. As described in the homonymous subsection of the Materials and Methods, the AC magnetic field was controlled to keep the samples at 43°C (treatment temperature) for 30 min. DOX was quantitated via HPLC before and after MHT, and the released DOX was calculated *via* Eq. 4, resulting in 47% for DPML and 46% for DFPML (Figure 3B). Supplementary Figure S5 brings a set of pictures taken from the size exclusion chromatography (SEC) undertaken for DOX-loaded nanocarriers (DPML and DFPML) before and after MHT. For the samples submitted to SEC after MHT, the separation between free (released) DOX (orangish color) and the nanocarriers (DPML and DFPML, brownish color, due to the entrapped MnFe_2_O_4_ NPs) is visibly noticeable (Supplementary Figure S5).

#### In Vitro Assays: Conventional and Temperature-Mediated DOX Release for Cytotoxic Activity

**FIGURE 4:**
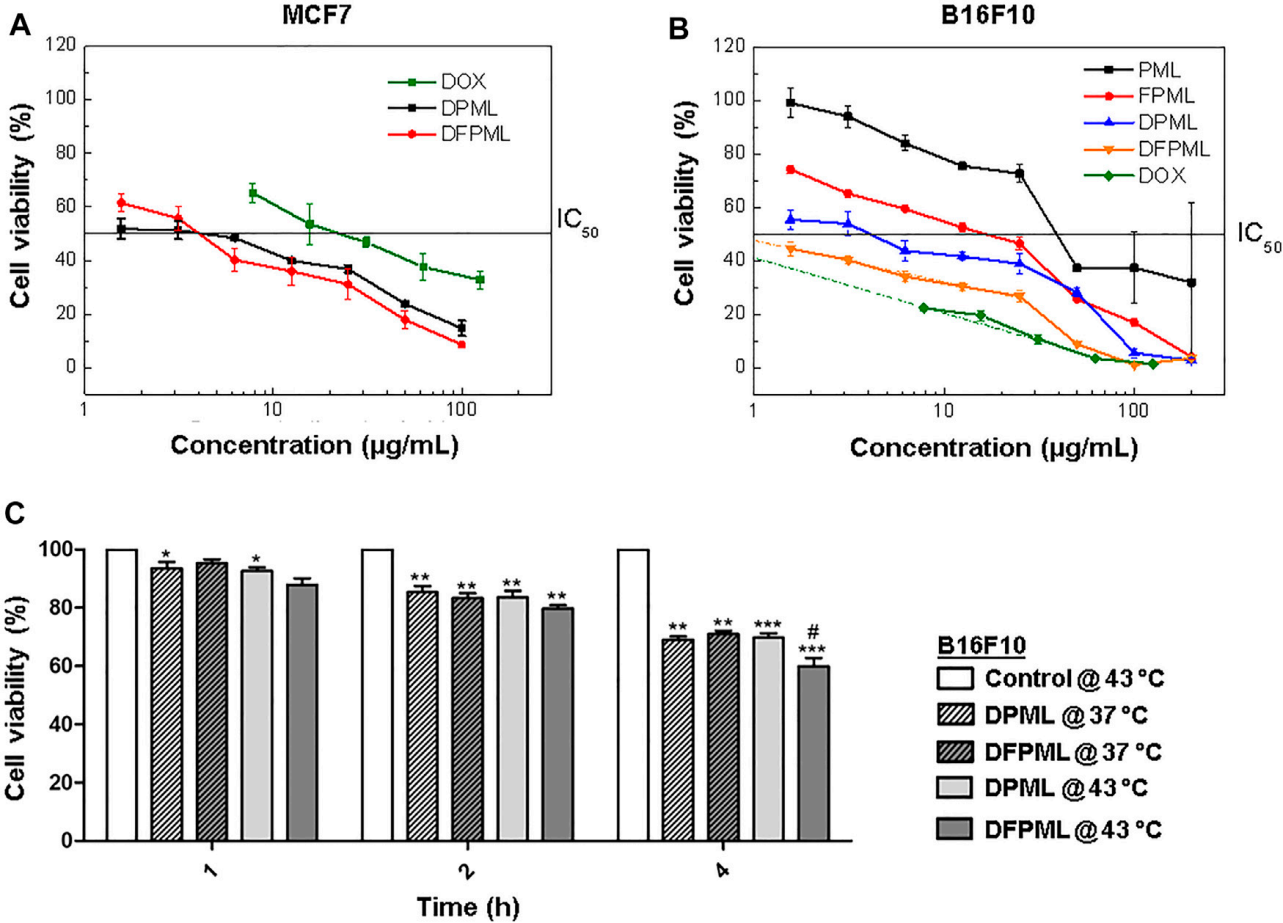
Conventional and temperature-mediated DOX release for cytotoxic activity. Cell viability as a function of nanocarrier concentration and IC_50_ determination for both **(A)** MCF7 human mammary adenocarcinoma and **(B)** B16F10 murine melanoma cells exposed to different control (DOX, PML, FPML, and DPML) and DFPML formulations. **(C)** Temperature-induced cytotoxicity of DPML and DFPML incubated with B16F10 cells under either 37°C (normothermia) or 43°C (hyperthermia).

Figure 4A shows the results for an *in vitro* cytotoxicity assay with MCF7 cells. Both DFPML and DPML exhibited similar half-maximal inhibitory concentrations (IC_50_), within the 4.2 μg/ml and 4.3 μg/ml range, five- to six-fold lower than the IC_50_ for free DOX.

In contrast, as shown in Figure 4B, IC_50_ values for control formulations without DOX were significantly higher for B16F10 cells: 50 μg/ml for PML and 12 μg/ml for FPML. On the other hand, the IC_50_ value for the non-folate targeted control sample DPML was found to be 4.9 μg/ml, similarly to the half-maximal cytotoxicity concentration range obtained for MCF7 cells (4.2–4.3 μg/ml). However, a six-fold lower IC_50_ value was obtained for DFPML, around 0.8 μg/ml, which is within the same order of magnitude of the IC_50_ found for free DOX (about 0.4 μg/ml).

Figure 4C shows the results for *in vitro* assays with B16F10 cells, where the cells were exposed to the previously determined IC_50_ concentrations of either DPML or DFPML but incubated either at 37°C (normothermia) or 43°C (hyperthermia) for 1, 2, 4 h. Time-dependent cytotoxicity was observed, with the number of viable cells decreasing in time for all of the tested formulations.

No direct temperature-induced cytotoxicity was verified, since the number of viable cells in control samples (not exposed to any of the DOX-loaded formulations) remained unchanged over time, both at normothermia (data not shown) and hyperthermia conditions (Figure 4C, white bars). In contrast, a significant influence of temperature over cytotoxicity could be observed over time for the test samples, especially for DFPML under hyperthermia conditions after 4 h of exposure (Figure 4C, non-crosshatched dark gray bar), suggesting an enhanced heat-induced DOX-release under these conditions.

## Discussion

In this study, we investigated the capabilities of manganese ferrite (MnFe_2_O_4_) magnetic nanoparticles (NPs) as the magnetothermal converting components of doxorubicin-loaded folate-targeted PEGylated magnetoliposomes (DFPML). Once submitted to controlled magnetic hyperthermia (MHT) conditions, these MnFe_2_O_4_ NPs release thermal energy due to dynamic hysteresis losses, which in turn mediates a controlled release of the chemotherapeutic agent doxorubicin (DOX). As reported extensively in the specialized literature, PEGylation confers stealth properties to liposomes nanocarriers as the DFPML used in this work (Napper, 1970; Antic et al., 2004; Urva et al., 2009), prolonging their half-life in blood and thus increasing the probability of the nanocarrier retention in tumors.

Moreover, the functionalization with folate moieties (FA), through the synthetic DSPE-PEG-FA molecule, enhances retention (and subsequently chemotherapeutic action) in folate receptor-rich areas. Hence, a non-invasive site-specific MHT-mediated therapy with DFPML could improve the treatment of tumors overexpressing folate receptors. Additionally, DFPML permits a significant decrease in the DOX dose needed for efficacious cytotoxicity, consequently minimizing the adverse effects of the treatment. Similarly, an analogous folate targeting strategy increased median survival times *in vivo* for acute myelogenous leukemia-bearing mice, as reported by Pan et al. (2002).

The final DFPML nanocarrier encapsulates dextran-coated MnFe_2_O_4_ NPs. However, attempts to encapsulate both citrate- and phosphate-coated MnFe_2_O_4_ NPs were also performed. In a previous work, we could successfully encapsulate stable citrate-coated MnFe_2_O_4_ NPs within both the aqueous core (pH ≈ 7) and lipidic phase of erythrocyte membrane-camouflaged magneto-fluorescent nanocarriers (Sousa-Junior et al., 2020). In the current study, DOX encapsulation requires an acidified liposome aqueous core (pH = 3) (Haran et al., 1993; Zucker et al., 2009). In this medium, both citrate- and phosphate-coated MnFe_2_O_4_ NPs could not form a stable colloidal dispersion, while dextran-coated MnFe_2_O_4_ NPs retained colloidal stability. This happens because decreasing pH for citrate- and phosphate-coated nanoparticles results in lower surface charge (zeta potential), therefore decreasing the repulsive electric interaction between the nanoparticles. It might also favor uncoating, and hence flocculation via van der Waals and magnetic attractive interactions. For the non-ionic dextran-coating, this effect is not observed, and colloidal stability is possible via steric repulsion (Napper, 1970).

MnFe_2_O_4_ NPs crystallinity could be observed both by TEM and XRD. From Figure 1B, the spacing between crystalline planes highlighted on the image could be estimated as 0.45 ± 0.02 nm. Since the unit cell parameter for a cubic manganese ferrite was reported as being a = 0.85 nm (Antic et al., 2004), if we consider the crystalline plane for which Miller’s indices are h = k = l = 1, the interplanar spacing $d_{111}=\sqrt{a^{2}/\left(h^{2}+k^{2}+l^{2}\right)}$ = 0.49 nm, in good agreement with our experimental estimation. Moreover, analyzing the XRD spectrum in Figure 1D, the NPs mean diameter could be estimated as 13.5 nm using Debye–Scherrer’s method. This value is also in very good agreement with the mean diameter determined via log-normal fit of the NP size distribution (14.4 ± 5.2 nm, Figure 1C). Moreover, the XRD results in Figure 1D match very well XRD results previously reported in the literature for cubic spinel MnFe_2_O_4_ NPs, which in turn are in good agreement with the standard diffraction values of JCPDS/ICDD file number 74–2403 (Ahmad et al., 2015; Mary Jacintha et al., 2017).

SLP values, derived for MnFe_2_O_4_ NPs at 4.5 mg/ml from Figure 1F, were proportional to the square of the applied AC magnetic field amplitude (Supplementary Figure S2), as expected for LRT (Linear Response Theory) conditions (Makridis et al., 2019). However, depending on the AC magnetic field amplitude and frequency ranges adopted, as well as on the sample concentration, NP size distribution and core material, and even on the experimental setup (adiabatic or non-adiabatic conditions), LRT might not be applicable (Verde et al., 2012; Soetaert et al., 2017). In this case, one might refer to non-LRT models (Carrião et al., 2017), and/or take other variables into account, such as magnetic dipolar interactions between NPs (Branquinho et al., 2013), or the fraction of blocked (non-superparamagnetic behaving) NPs in the sample (Aquino et al., 2019).

Also of note is that neither the MnFe_2_O_4_ NPs encapsulation within liposomes nor the subsequent functionalization of these liposomes with folate moieties and DOX cargo significantly affected their magnetic properties and magnetothermal conversion efficiencies (SLP). Indeed, comparing the VSM results for non-encapsulated MnFe_2_O_4_ NPs (Figure 1E) and those for DPML and DFPML (Figure 2E), no significant differences were observed in terms of their magnetic behavior. Additionally, all magnetoliposomes prepared for this study (PML, FPML, DPML, and DFPML) displayed similar SLP values (56 W/g in average, see Figure 2F and Supplementary Table S2), which were in turn very close to the SLP value obtained for free MnFe_2_O_4_ NPs submitted to the same MHT conditions (about 60 W/g, see Figure 1F and Supplementary Table S1, for a 342 Oe and 334 kHz AC magnetic field). Nevertheless, the slight decrease in the SLP values observed after their entrapment might be explained by increased interparticle interactions (impacting Néel-Brown relaxation times) and/or decreased mobility (impacting Brown relaxation times) as a result either of their confinement, or of a decrease of their equilibrium susceptibility (Branquinho et al., 2013; Di Corato et al., 2014; Aquino et al., 2019).

As a consequence of this non-significant impact on the MnFe_2_O_4_ NPs performance under MHT, MHT-mediated DOX release from both DPML and DFPML samples were also very similar (Figure 3). The DOX load released from these nanocarriers in 30 min (46 and 47%, respectively) suggests that controlled release of the therapeutic cargo could be successfully implemented. Similar results (about 45% of the DOX cargo released by magnetoliposomes after 1800 s of MHT) were reported by Joniec et al. (Joniec et al., 2016). Skouras et al. emphasize that co-encapsulation of DOX and magnetic NPs within liposomes does not affect DOX loading/retention (Skouras et al., 2018). After 6 days under no external magnetic excitation, Askari et al. reported that about 50% of the DOX cargo is still retained within their magnetoliposomes, an evidence of the stability of these nanosystems (Askari et al., 2020).

Like Doxil^®^, DFPML is a PEGylated liposome, DOX-loaded via a transmembrane ammonium sulfate gradient method (Barenholz, 2012a). Unlike Doxil^®^, however, DFPML can release its DOX cargo in a non-pH-dependent remotely-controlled manner, via a non-invasive locally-applied AC magnetic field. For hydrophobic magnetic NPs, mainly entrapped within the liposome lipid bilayer, DOX release is associated with the bilayer rupture, induced by the NPs mechanical vibration (Joniec et al., 2016). On the other hand, for hydrophilic magnetic NPs, mainly entrapped within the liposome aqueous cavity, DOX release is associated with changes in the bilayer permeability (pore formation), induced by the thermal energy released by the magnetically excited NPs (Ponce et al., 2006). Worthy of note, DOX release from ThermoDox^®^ (Celsion Corp.) relies on its thermoresponsive bilayer, composed of thermosensitive lipids (Needham et al., 2000; Dunne et al., 2017), whereas DOX release from DFPML relies on heat-induced bilayer permeability changes, even though the bilayer is composed of non-thermosensitive lipids.

Finally, our *in vitro* results with tumor cell strains suggest that DFPML display higher cytotoxic activity at 43°C, as a result of an increased DOX release (Figure 4B). DFPML outperformed DPML in terms of cytotoxicity, especially for longer exposure times, suggesting that the folate-targeting strategy did enhanced cytotoxicity for folate receptor-expressing B16F10 cells (Gabizon et al., 2010; Gazzano et al., 2018). DPML and DFPML outperformed both the DOX-loaded magnetoliposomes developed by Rȩkorajska et al. (Rȩkorajska et al., 2019) and Askari et al. (Askari et al., 2020), respectively with a 2-fold and a 3.5-fold lower IC_50_ value for MCF7 cells (Figure 4A). The even lower IC_50_ value for B16F10 (Figure 4B) suggests that B16F10 cells overexpress folate receptors compared to MCF7 cells. Curiously, Battogtokh and Ko (Battogtokh and Ko, 2016) did not observe significant cytotoxicity differences between these cell lines for different formulations. The apparent discrepancy between the aforementioned results and ours might be explained by the longer exposure time (6 h) adopted by the aforementioned authors as well as by the combination with photodynamic therapy (PDT).

## Conclusion

In previous studies, our group reported MHT-mediated controlled release of drugs from polymeric nanospheres and nanocapsules (Oliveira et al., 2012), as well as from solid lipid nanoparticles (SLN) (Oliveira et al., 2018). We have also previously studied the physicochemical properties and the encapsulation efficiency of magnetic NPs within liposomes, forming magnetoliposomes (Cintra et al., 2009; Salvador et al., 2016). More recently, we have reported the co-encapsulation of paclitaxel and imatinib within folate-targeted liposomes (Peres-Filho et al., 2018). In this study, we combine the knowledge acquired from these previous works, successfully accomplishing the MHT-mediated controlled release of doxorubicin from folate-targeted PEGylated magnetoliposomes (DFPML). The folate targeting proved to be a successful strategy, with DFPML being more cytotoxic for folate receptor-overexpressing B16F10 cells, compared to non-folate targeted control formulations. In a near future, we intend to explore other heat-mediated mechanisms of controlled release, both *in vitro* and *in vivo*, for instance, near-infrared (NIR) photo-hyperthermia (PHT) mediated by photothermal converting agents (Li et al., 2019), such as gold NPs (Qin and Bischof, 2012; Fang and Chen, 2013) or even magnetic NPs (Jaque et al., 2014; Sousa-Junior et al., 2020).

## Data Availability

The original contributions presented in the study are included in the article/Supplementary Material. Further inquiries can be directed to the corresponding author.
