# Supplementary material for: Folate-Targeted PEGylated Magnetoliposomes for Hyperthermia-Mediated Controlled Release of Doxorubicin
Source: Front Pharmacol. 2022 Mar 21;13:854430. doi: 10.3389/fphar.2022.854430 (PMC8978894; doi:10.3389/fphar.2022.854430)
Supplement: Supplementary file 1 [file DataSheet1.docx]

***Supplementary Material***

**Figure S1** – Magnetization profile for MnFe_2_O_4_ NPs (powder)


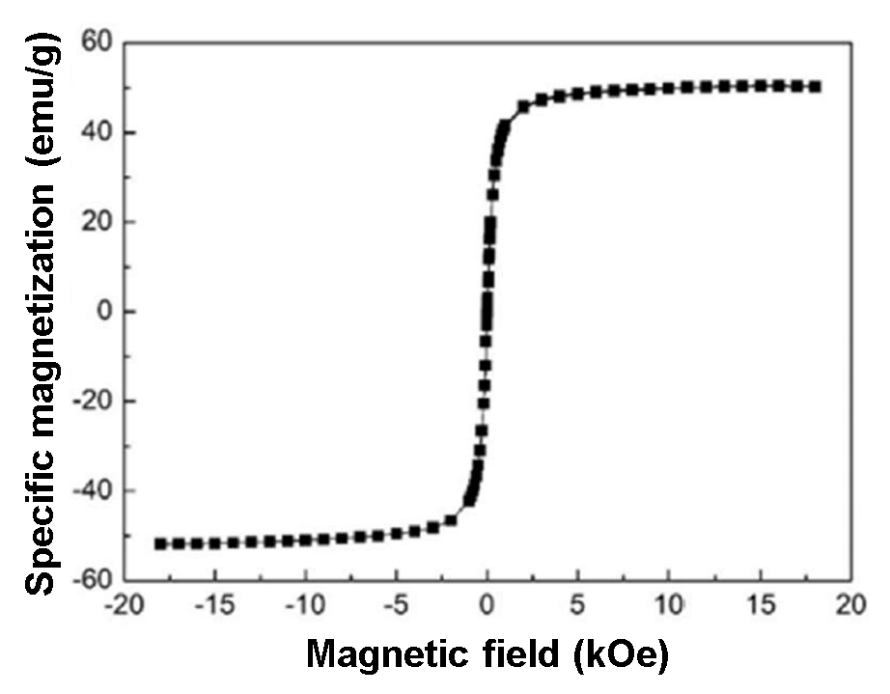


**Figure S2 –** SLP × H for MnFe_2_O_4_ NPs


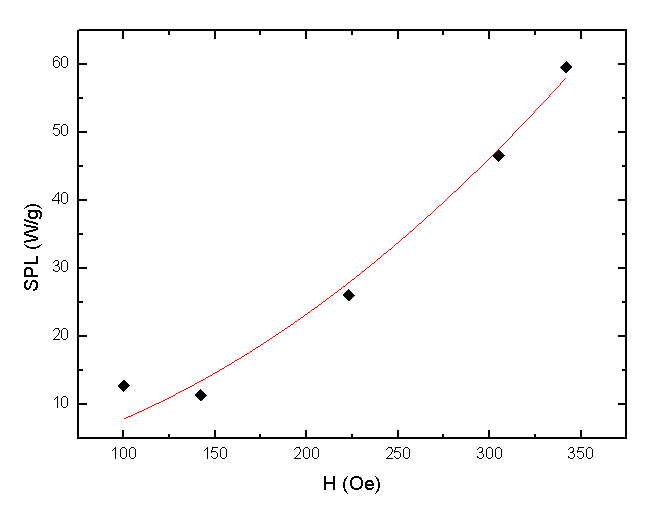


**Figure S3 –** FTIR for DSPE-PEG


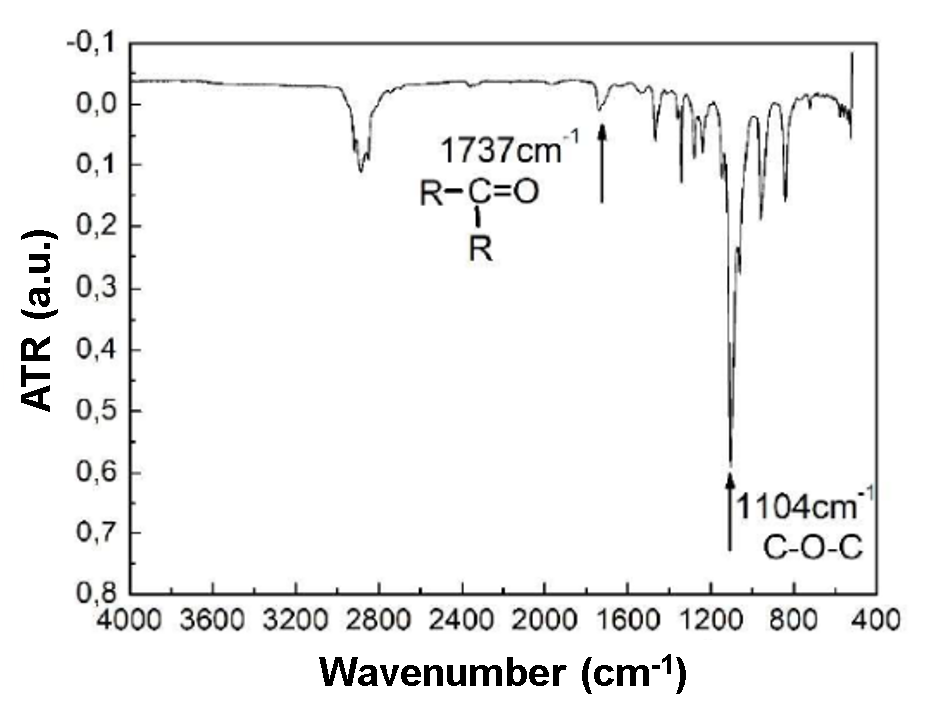


**Figure S4 –** FTIR for folic acid


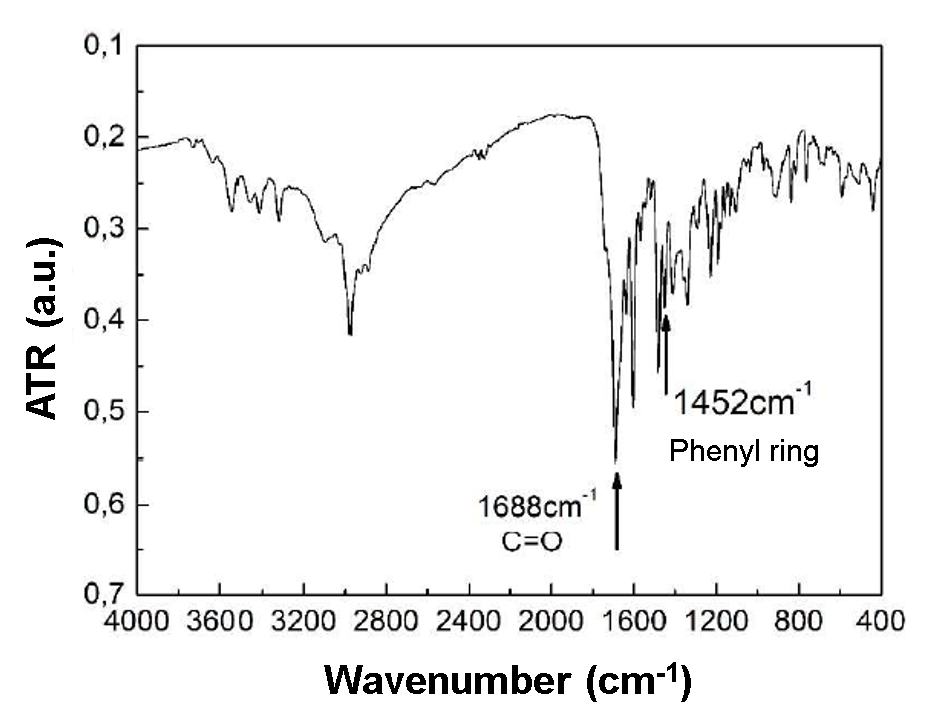


**Figure S5 –** SEC for DOX-loaded magnetoliposomes before and after MHT


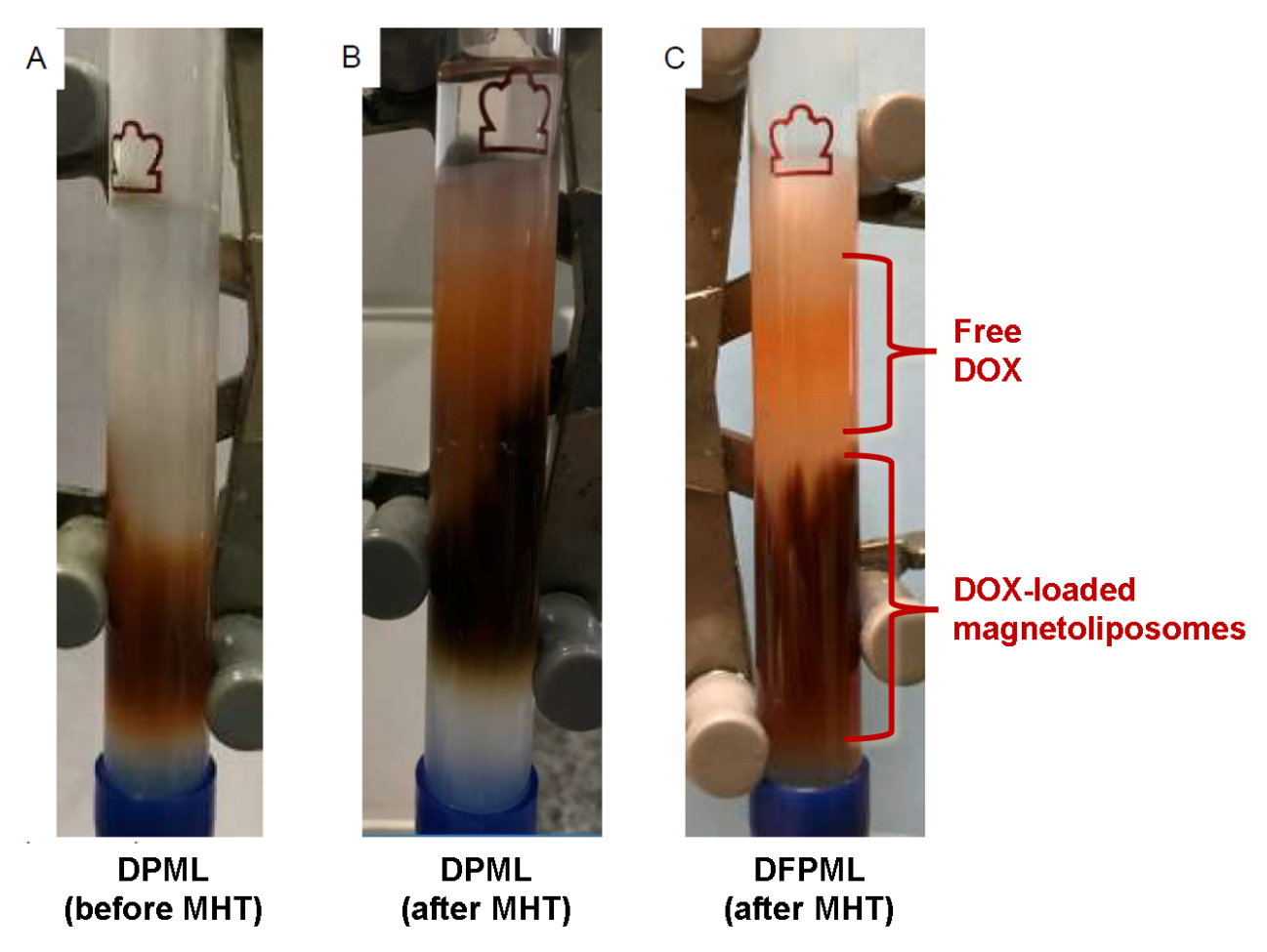


**Table S1 –** SLPs for different AC magnetic field amplitudes

| **Field (Oe)** | **SLP (W/g)** |
| --- | --- |
| 100 | 12.8 |
| 142 | 11.4 |
| 223 | 26.1 |
| 305 | 46.6 |
| 342 | 59.6 |

**Table S2 –** SLPs for PML, FPML, DPML, and DFPML under AC magnetic field (342 Oe, 334 kHz)

| **Formulation** | **SLP (W/g)** |
| --- | --- |
| PML | 56.1 |
| FPML | 55.4 |
| DPML | 57.3 |
| DFPML | 54.9 |
